# Downregulation of Homologous Recombination DNA Repair Genes by HDAC Inhibition in Prostate Cancer Is Mediated through the E2F1 Transcription Factor

**DOI:** 10.1371/journal.pone.0011208

**Published:** 2010-06-18

**Authors:** Sushant K. Kachhap, Nadine Rosmus, Spencer J. Collis, Madeleine S. Q. Kortenhorst, Michel D. Wissing, Mohammad Hedayati, Shabana Shabbeer, Janet Mendonca, Justin Deangelis, Luigi Marchionni, Jianqing Lin, Naseruddin Höti, Johan W. R. Nortier, Theodore L. DeWeese, Hans Hammers, Michael A. Carducci

**Affiliations:** 1 Prostate Cancer Program, The Sidney Kimmel Comprehensive Cancer Center at Johns Hopkins, Johns Hopkins University School of Medicine, Baltimore, Maryland, United States of America; 2 Department of Radiation Oncology and Molecular Radiation Sciences, Johns Hopkins University School of Medicine, Baltimore, Maryland, United States of America; 3 Division of Medical Oncology, Leiden University Medical Center, Leiden, The Netherlands; University Medical Center Hamburg-Eppendorf, Germany

## Abstract

**Background:**

Histone deacetylase inhibitors (HDACis) re-express silenced tumor suppressor genes and are currently undergoing clinical trials. Although HDACis have been known to induce gene expression, an equal number of genes are downregulated upon HDAC inhibition. The mechanism behind this downregulation remains unclear. Here we provide evidence that several DNA repair genes are downregulated by HDAC inhibition and provide a mechanism involving the E2F1 transcription factor in the process.

**Methodology/Principal Findings:**

Applying Analysis of Functional Annotation (AFA) on microarray data of prostate cancer cells treated with HDACis, we found a number of genes of the DNA damage response and repair pathways are downregulated by HDACis. AFA revealed enrichment of homologous recombination (HR) DNA repair genes of the BRCA1 pathway, as well as genes regulated by the E2F1 transcription factor. Prostate cancer cells demonstrated a decreased DNA repair capacity and an increased sensitization to chemical- and radio-DNA damaging agents upon HDAC inhibition. Recruitment of key HR repair proteins to the site of DNA damage, as well as HR repair capacity was compromised upon HDACi treatment. Based on our AFA data, we hypothesized that the E2F transcription factors may play a role in the downregulation of key repair genes upon HDAC inhibition in prostate cancer cells. ChIP analysis and luciferase assays reveal that the downregulation of key repair genes is mediated through decreased recruitment of the E2F1 transcription factor and not through active repression by repressive E2Fs.

**Conclusions/Significance:**

Our study indicates that several genes in the DNA repair pathway are affected upon HDAC inhibition. Downregulation of the repair genes is on account of a decrease in amount and promoter recruitment of the E2F1 transcription factor. Since HDAC inhibition affects several pathways that could potentially have an impact on DNA repair, compromised DNA repair upon HDAC inhibition could also be attributed to several other pathways besides the ones investigated in this study. However, our study does provide insights into the mechanism that governs downregulation of HR DNA repair genes upon HDAC inhibition, which can lead to rationale usage of HDACis in the clinics.

## Introduction

Epigenetic regulation of gene expression is thought to be brought about by both chromatin modulators that modify N-terminal tails of histones and DNA methylating enzymes that methylate CpG clusters in the promoter regions of eukaryotic genomes [1], [2], [3]. Cancer cells modulate the epigenetic machinery to silence tumor and metastatic suppressors to gain selective growth and invasive properties [4], [5], [6]. The HDAC class I and class II enzymes form complexes with co-repressors such as NuRD and the SMRT/NCoR complexes [7]. Cancer cells, including prostate cancer (PCa), recruit different HDACs associated with these large multi-protein co-repressor complexes to silence tumor suppressor genes and this serves as one rationale for the use of HDACis to treat cancer [8], [9].

The activity of both class I and class II HDACs is inhibited by short chain fatty acids (Phenylbutyrate, Valproic acid (VPA)) and hydroxamic acids (Vorinostat, Trichostatin A), while benzamides (MS-275) appear to be specific to Class I HDACs [8]. Conversely, class III HDACs, the sirtuins, are not inhibited by any of these agents [10]. Recently, Vorinostat has been approved by the FDA for the treatment of cutaneous T cell lymphoma. We and others have shown that treatment of PCa with HDACis or DNA methyltransferase inhibitors relieves the repression, causing reexpression of silenced tumor suppressors leading to cell cycle arrest, senescence and apoptosis [11], [12], [13]. The combination of HDACis with other agents has been shown to be effective for a wide variety of cancers. Although HDACis have been known to upregulate a number of genes, paradoxically an equal number of genes are repressed upon HDAC inhibition [14], [15], [16]. Repression of genes upon HDAC inhibition can be the result of indirect actions of repressors that are activated and cause repression in an HDAC passive fashion, or repression could be brought about by active recruitment of HDACs to promoters of selected genes [17]. Pathways that are downregulated upon HDAC inhibition create settings for treatment modalities that are ineffective in their presence. Recent reports suggest that HDACis such as phenyl butyrate, VPA, MS-275 and SAHA can potentiate radiation sensitivity of cancer cells [18], [19], [20], [21]. Transcriptional downregulation of certain genes involved in the homologous recombination (HR) and non-homologous end joining (NHEJ) DNA repair pathways have been implicated [18], [19], [20], [22].

Double strand breaks (DSBs) can be induced by endogenous agents such as reactive oxygen species and replication stress by stalled replication forks, or can be induced by exogenous agents like ionizing radiation [23]. It is increasingly evident that DNA damage is sensed by protein complexes, termed DNA damage sensors, which in turn induce a signal transduction cascade that recruit mediator and effector proteins to the damaged sites, leading to the repair of DNA [24]. Depending upon the extent of damage, further signal transduction alerts the cell to either delay the cell cycle through checkpoint activation for repair processes to complete, or undergo apoptosis [24]. Each type of DNA damage is sensed and repaired by distinct DNA repair pathways. The MRN complex, consisting of Mre11-Rad50-NBS1 mediator complex, senses DSBs and recruits ATM, a PI3K-like kinase, to the site of DSBs [25]. ATM is activated after recruitment to DSBs and phosphorylates downstream substrates, initiating the signal transduction process [26].

ATM and related kinases, ATR and DNAPK, phosphorylate a histone variant γ-H2AX at ser-139 which loads to sites of DSBs and covers megabases flanking the DSBs; this constitutes the irradiation induced foci. Phosphorylated γ-H2AX recruits several mediator proteins [27]. Among these mediators are the BRCA1 associated surveillance complex and 53BP1. The signal transduction cascade is further amplified by transducer checkpoint kinases, CHK1 and CHK2, which are activated upon phosphorylation by ATM and ATR. ATM and ATR together with CHK1/CHK2 kinases phosphorylate a number of effector substrates that include BRCA1, Rad51, p53, and Mdm2 [28]. Phosphorylation of p53 leads to its stabilization, causing a cell cycle arrest through induction of p21, or in the event of greater DNA damage, apoptosis. Stalled replication forks that arise due to DNA damage and replication stress, are sensed by PCNA related Rad9-Rad1-Hus1 or the 911 complex, whose recruitment to the stalled site is mediated by replication protein A and ATR. The final effector proteins that repair the damaged DNA are activated and recruited by the above mentioned kinases and mediator proteins to the site of damage and repair ensues.

Genes of the DNA repair pathways are tightly regulated both at the transcriptional and post transcriptional level. At the transcriptional level, the E2F transcription factors have been known to play a role in regulating repair proteins. There are nine known E2F transcription factors that can be divided into transcriptional activators (E2F1, E2F2, E2F3a) and repressors (E2F4-8); E2F3b has been hypothesized to function as a repressor [29]. E2F1 and E2F4 have been implicated in the regulation of CHK1, BRCA1 and RAD51 genes [30], [31], [32].

Recently, we reported a novel “multiple-loop, double-cube” cDNA microarray design, to analyze HDAC inhibitor induced changes in gene expression across sensitive and resistant PCa cell lines [16]. Applying Analysis of Functional Annotation (AFA) on the data set from the above microarray experiment we found that several genes of the HR repair and DNA damage response pathway are downregulated upon HDAC inhibition. In this report, we demonstrate transcriptional downregulation of several DNA damage response genes in PCa cells upon HDAC inhibition, provide functional evidence of the involvement of HR repair pathway in compromised DNA repair, and provide a role of the E2F1 transcription factor in the downregulation of DNA damage response genes.

## Results

### AFA reveals HDACis downmodulate several genes of the DNA damage and response pathway in PCa

**Figure 1:**
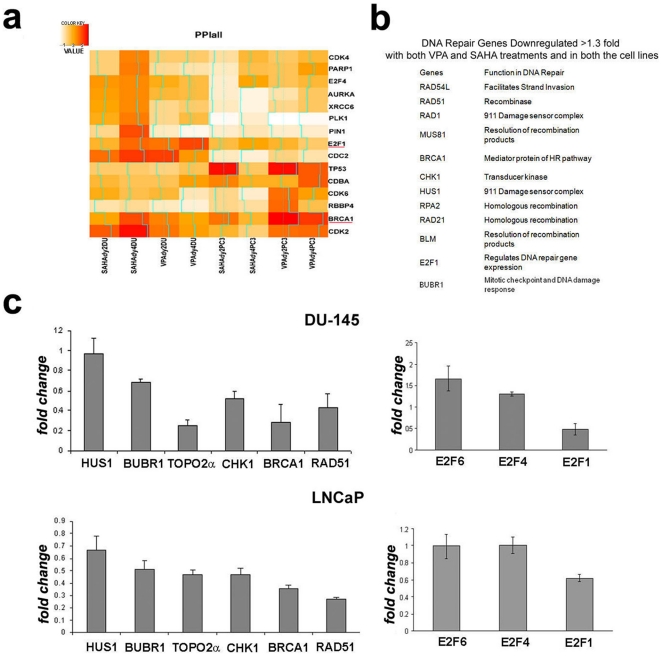
Analysis of Functional Annotation (AFA) in HDACi treated PCa cells. a) DU145 and PC3 cells were treated with two different HDACis (vorinostat (SAHA, 1 µM), and VPA (Valproic acid, 1 mM) for incubation periods (2days and 4days). AFA reveals down-regulation of genes involved with DNA damage and response (see Table 1). AFA results for protein-protein interaction indicate BRCA1 and E2F interacting networks are affected by HDAC inhibition. Color code represents as 10^-n^ the *p-*values obtained from the Wilcoxon rank-sum test. b) DNA repair genes downregulated ≥1.3 fold in both PC3 and DU-145 cells upon treatment with both VPA and vorinostat. c) Validation of the AFA was done by a Q-PCR analysis on a subset of genes downregulated upon 1.5 mM VPA treatment. Results are depicted as fold change over untreated control cells.

AFA was performed on our recently published microarray data set of two PCa cell lines (PC3 and DU-145) treated with vorinostat (previously known as SAHA) and VPA [16]. The analysis revealed downregulation of several genes involved with DNA damage response and repair (Table 1). Protein-protein interaction analysis on the microarray data revealed several genes related to the E2F1 and BRCA1 pathways were downregulated with both HDACis more than 1.3 fold in both PCa cells lines (Fig. 1a). Many of these downregulated genes are involved in the HR DNA repair pathway. Surprisingly, the NHEJ pathway related genes, specifically DNAPK and Ku, were not affected in our arrays. Earlier reports have shown that RAD51 is downregulated upon HDAC inhibitor treatment in a variety of cell lines [18], [19], [20]. Our microarray data revealed that besides Rad51 and related genes, a wide variety of genes involved in the DNA damage response and repair pathway such as Brca1, Chk1, Topo IIα, Hus1, and Bubr1, were downregulated upon HDAC inhibitor treatment (Fig. 1b).

**Table 1:** Analysis of Functional Annotation results for gene ontology.

				VPA		SAHA	
GO identifier	Ontology	GO Term	Time	PC3	DU145	PC3	DU145
**GO:0000077**	**BP**	**DNA damage checkpoint**	**48 hs**	>0.10000	0.06593	>0.10000	>0.10000
			**96 hs**	>0.10000	>0.10000	0.04165	>0.10000
**GO:0003684**	**MF**	**damaged DNA binding**	**48 hs**	0.04926	0.01893	>0.10000	0.00537
			**96 hs**	0.00329	0.01312	>0.10000	0.03314
**GO:0006974**	**BP**	**response to DNA damage stimulus**	**48 hs**	<0.00001	<0.00001	0.03633	<0.00001
			**96 hs**	<0.00001	<0.00001	<0.00001	<0.00001
**GO:0042770**	**BP**	**DNA damage response, signal transduction**	**48 hs**	0.07854	0.01562	0.04007	>0.10000
			**96 hs**	>0.10000	0.04847	0.03714	>0.10000
**GO:0006281**	**BP**	**DNA repair**	**48 hs**	<0.00001	<0.00001	>0.10000	<0.00001
			**96 hs**	<0.00001	<0.00001	0.00007	<0.00001
**GO:0006302**	**BP**	**double-strand break repair**	**48 hs**	0.08622	0.00008	>0.10000	0.00437
			**96 hs**	0.02245	0.00011	>0.10000	0.01144
**GO:0006284**	**BP**	**base-excision repair**	**48 hs**	0.00182	0.00510	0.02607	0.00442
			**96 hs**	0.00031	0.00204	0.08656	0.00286
**GO:0006298**	**BP**	**mismatch repair**	**48 hs**	>0.10000	>0.10000	>0.10000	>0.10000
			**96 hs**	0.05092	>0.10000	>0.10000	>0.10000
**GO:0006289**	**BP**	**nucleotide-excision repair**	**48 hs**	>0.10000	>0.10000	>0.10000	>0.10000
			**96 hs**	0.07408	>0.10000	>0.10000	>0.10000
**GO:0000724**	**BP**	**double-strand break repair via homologous recombination**	**48 hs**	>0.10000	0.01723	>0.10000	0.03906
			**96 hs**	>0.10000	0.01551	>0.10000	0.09229
**GO:0000725**	**BP**	**recombinational repair**	**48 hs**	>0.10000	0.01723	>0.10000	0.03906
			**96 hs**	>0.10000	0.01551	>0.10000	0.09229
**GO:0045739**	**BP**	**positive regulation of DNA repair**	**48 hs**	>0.10000	>0.10000	>0.10000	0.07948
			**96 hs**	>0.10000	>0.10000	>0.10000	0.07583
**GO:0006310**	**BP**	**DNA recombination**	**48 hs**	0.01415	0.00001	>0.10000	0.00107
			**96 hs**	0.00036	<0.00001	>0.10000	0.00009

The enrichment driven by down-regulation of gene expression showed an overall involvement of processes related to the response of DNA damage. The reports the P-values obtained from the Wilcoxon rank-sum test after correction for multiple testing by the Benjamini-Hochberg method.

In order to understand the consequence of downmodulation and gain more insights into the mechanism behind the downmodulation, we performed further analysis using two PCa cell lines (DU-145 and LNCaP) using the HDAC inhibitor VPA. To validate our microarray data, we performed a Q-PCR analysis on a subset of repair genes. Our data revealed that all the genes tested were downregulated in both of the cell lines, except Hus1 which was downregulated only in LNCaP cells (Fig. 1c). BRCA1, Rad51 and Chk1 are known to be regulated by the E2F transcription factor [30], [31]. Since the AFA revealed enrichment for downmodulation of E2F1 target genes, including E2F1 itself, we investigated the transcript level of both the activator (E2F1) and repressor E2F (E2F4 and 6) transcription factors. Our results show that E2F1 was significantly downregulated in both the cell lines treated with VPA, while the repressor E2Fs were not affected in either of the cells lines upon VPA treatment (Fig. 1c).

### Downmodulation of DNA repair genes by HDAC inhibition leads to an increased sensitivity of PCa cells to DNA damaging agents

**Figure 2:**
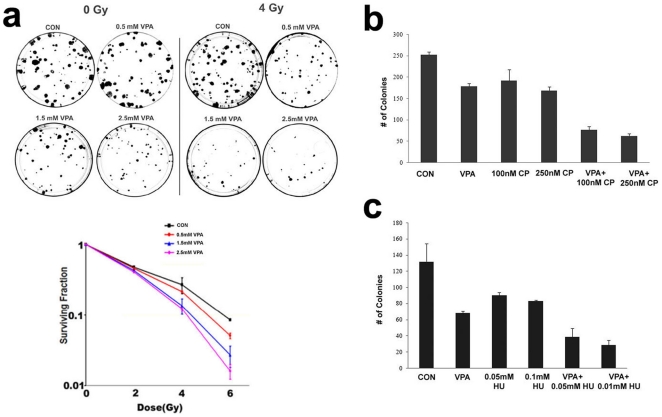
Sensitivity of DU-145 cells to DNA damaging agents upon VPA treatment. a) Clonogenic assay performed in DU-145 after treatment with different doses of VPA for 48 h before irradiation with different doses of radiation. Top panel shows representative clonogenic plates with 0Gy and 4 Gy radiation graph below depicts surviving fraction after VPA treatments and irradiation. Error bar represents standard deviation of three independent experiments. b) Clonogenic assay performed on DU-145 cells treated with 1.5 mM VPA and cisplatin (100 nM and 250 nM) for 48 h. Error bar represents standard deviation. c) Clonogenic assay performed on DU-145 cells treated with 1.5 mM VPA and hydroxyurea (0.5 mM and 1 mM) for 48 h. Error bar represents standard deviation.

Previous reports from our group and others have demonstrated that HDACis like VPA can decrease proliferation of prostate cancer cells[11], [16]. HDACis have also been known to act as radiosensitizers [18], [19], [20]. We hypothesized that downmodulation of DNA repair genes upon HDAC inhibition would lead to an increased sensitivity to various DNA damaging agents. DU-145 cells were subjected to clonogenic survival assays after treatment with a combination of HDACis and different agents that induce DSBs such as radiation, cisplatin and hydroxyurea. A radiosensitivity clonogenic assay was performed with increasing dosages of VPA in the presence of an increasing dosage of radiation. As expected, VPA did radiosensitize DU-145 cells, and there was an increase in sensitivity with increasing dosages (Fig. 2a). Recently, it has been demonstrated that a defect in homologous recombination can lead to changes in drug sensitivity profile, rendering the BRCA1 deficient breast cancers sensitive to mitomycin C, cisplatin, etoposide and other drugs that produce double-stranded lesions [33]. We argued that this may be true for HDACi treated PCa cells, where there is a decrease in BRCA1 pathway related gene expression. Clonogenic assays performed after treatment of DU-145 cells with VPA alone or in combination with cisplatin and hydroxyurea, revealed that in comparison to a single agent, the combination of VPA with either hydroxyurea or cisplatin greatly decreased clonogenic survival (Fig. 2b and c).

### HDAC inhibition by VPA leads to a decrease in DNA repair proteins of the HR and DNA Damage Response pathway

**Figure 3:**
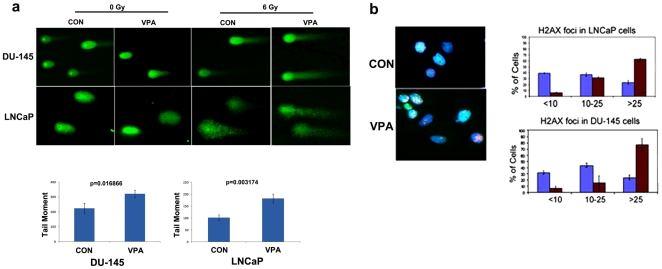
VPA treated prostate cancer cells show decreased repair capacity and γ-H2AX clearance. a) Neutral comet assay performed on prostate cancer cells treated with 1.5 mM VPA for 48 h and irradiated with 6 Gy γ- radiation followed by a 4 h repair interval. Unirradiated cells (0Gy) with and without VPA treatment did not show any significant difference in comet tail moments. The graph depicts average tail moment of 50 cells error bar indicates SD value of three experiments. Comparisons have been performed using the student's t-test. b) Immunofluorescence showing H2AX Ser^139^ staining in DU-145 cells treated with 1.5 mM VPA for 48 h and irradiated with 4Gy radiation followed by 4 h repair time. The graph shows quantitation of H2AX foci in control (blue column) and treated (red column) DU-145 and LNCaP cells. Error bar represents standard deviation of three independent experiments.

**Figure 4:**
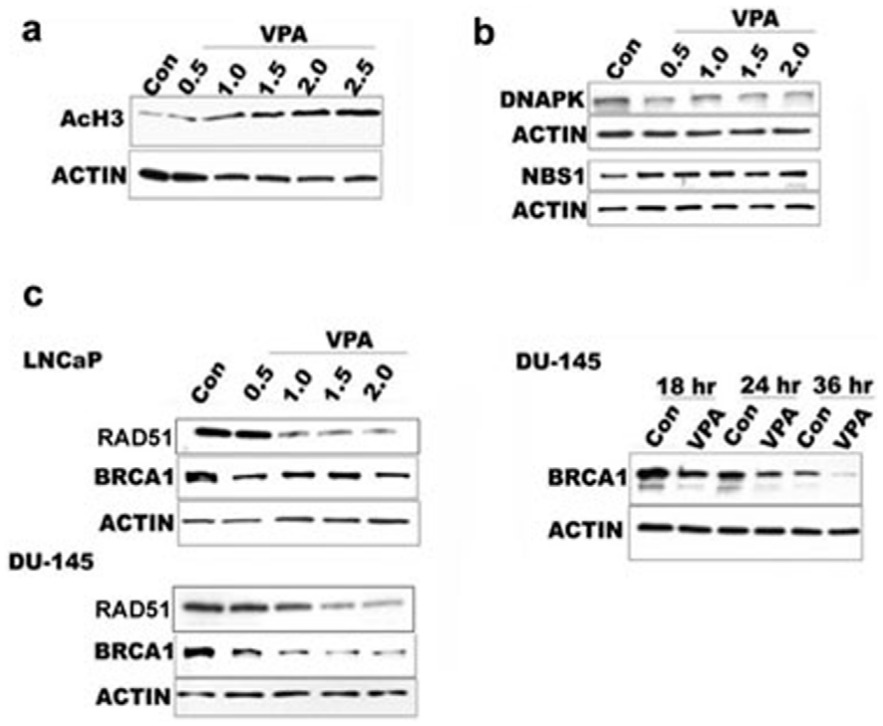
Repair protein in prostate cancer cells treated with VPA. a) Western blot showing acetylation of histone H3 protein in DU-145 cells after treatment with different doses of VPA for 48 h. b) DNAPK and NBS1 protein levels in VPA treated DU-145 cells for 48 h. c) Western blot showing RAD51 and BRCA1 protein levels in LNCaP and DU-145 cells treated with varying dosage of VPA for 48 h. Blot on the right shows DU-145 cells treated with 1.5 mM VPA for varying timepoints probed for BRCA1 protein.

**Figure 5:**
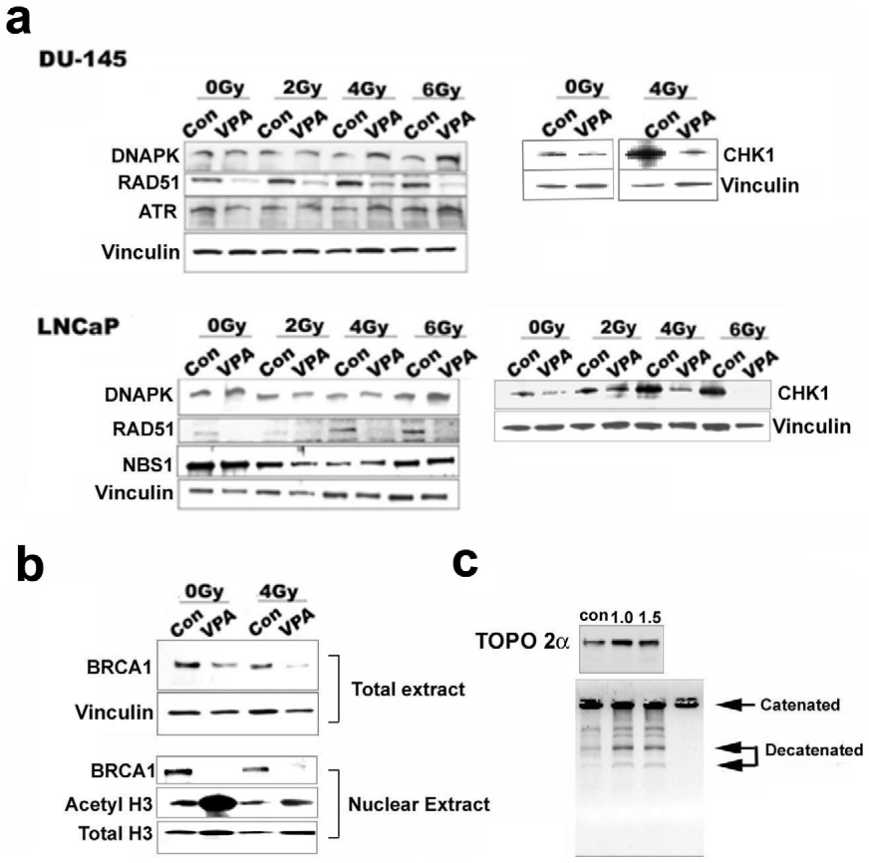
Repair protein levels in VPA treated PCa cells followed by irradiation. a) PCA cell lines treated with 1.5 mM VPA for 48 h, irradiated with different doses of radiation, probed for various repair proteins by western blotting. Unirradiated (0Gy cells) were also included. b) Total and nuclear extract of VPA (1.5 mM for 48 h) treated DU-145 cells with and without irradiation probed for BRCA1 protein. Unirradiated (0Gy cells) were also included. c) Top panel shows TOPO IIα protein level in nuclear extract from DU-145 cells upon VPA treatment. Bottom panel is an agarose gel showing TOPO IIα activity in the same extracts, lane 4 is a negative control.

Sensitivity to various DNA damaging agents upon HDAC inhibition could be the result of compromised or subnormal DNA repair ability in treated cells. This can be brought about by the downregulation of DNA repair genes upon HDAC inhibition. Radiosensitization of HDACis has been linked to a decrease in Rad51 gene expression in PCa [20]. To first test whether VPA causes a decrease in double strand break DNA repair capacity of PCa cells we performed a neutral comet assay to assess for DNA repair ability of prostate cancer cells upon HDAC inhibition [34], [35]. Under the experimental conditions used, we did not find any statistical difference in the tail moment of VPA treated and untreated control cells without radiation. However, in the absence of VPA treatment cells exposed to 4 Gy of irradiation were able to repair most of their damaged DNA within 4 hours. However after VPA treatment both prostate cell lines showed significantly higher tail moments 4 hours after exposure to 4 Gy of irradiation suggesting reduced DNA repair capacity (Fig. 3a). To determine whether the DSB repair is compromised upon HDAC inhibition, we employed H2AX foci clearance as an indicator of efficient DSB repair. DU-145 and LNCaP cells were treated with VPA and irradiated with 2 Gy, 4 Gy, and 6 Gys of radiation. Cells were fixed after 4 h of repair and probed with Ser^139^ phosphorylated H2AX antibodies. As expected, an increase in H2AX foci was found in PCa cells treated with VPA, indicating a decrease in DNA repair capacity (Fig. 3b). Compromised DNA repair can be the result of a decrease in the total amounts of repair protein and/or a decrease in localization or recruitment of repair proteins to the damaged site. To test these possibilities, we first investigated the levels of repair proteins that were shown to be downmodulated in our microarray dataset after VPA treatment. Many of these genes, such as Rad51, Brca1 and Chk1, are induced upon DNA damage [24]. To investigate whether these proteins remain downregulated upon HDAC inhibition even upon DNA damage, we performed our analysis in the absence and presence of radiation. An increase in total H3 acetylation in DU-145 and LNCaP cells demonstrated that VPA does cause an effective global HDAC inhibition at the dosage used for the experiments (Fig. 4a and data not shown). Increasing the concentration of VPA causes a decrease in BRCA1 and Rad51 in both DU-145 and LNCaP cells, while other repair proteins such as DNAPK and NBS1 remain unaffected (Fig. 4b and c). BRCA1 protein levels are not steady in the cell, and peaks at different time points depending on the phase of the cell cycle. In order to understand when BRCA1 is downregulated in VPA treated cells, lysates were collected at each time point after treatment. BRCA1 was found to decrease as early as 18 h after treatment (Fig. 4c), indicating a rapid response upon HDAC inhibition. Irradiation of PCa cells, post treatment with VPA, demonstrated certain DNA damage response and repair proteins such as ATR and NBS1 remained unaffected, while DNAPK was induced upon VPA treatment in the presence of radiation (Fig. 5a). RAD51, BRCA1 and CHK1 were found to remain downregulated even upon irradiation in VPA treated cells. BRCA1 is a nuclear protein, which distributes to the cytoplasm under certain conditions. To ascertain that treatment with VPA results in the downregulation of BRCA1 in the nuclear compartment, DU-145 cells were treated with VPA and irradiated 48 h posttreatment with 4 Gy of radiation. Nuclear extracts prepared from these cells were immunoprobed for BRCA1. As shown in Fig. 5b, VPA treated cells have a downregulation of BRCA1 protein even after irradiation.

Our results had indicated a marked reduction in TOPO IIα transcript levels upon treatment with VPA. TOPO IIα resolves catenated DNA by inducing a transient DSB and subsequent religation [36]. TOPO IIα has been implicated in a variety of cellular processes including DNA replication, transcription and chromosome segregation [37]. We expected downregulation of the TOPO IIα protein after HDAC inhibition. To our surprise, we found TOPO IIα protein is upregulated upon VPA treatment in both prostate cell lines (Fig. 5c and data not shown). To investigate whether this leads to an increase in activity of the TOPO IIα protein, we used nuclear extract from VPA treated DU-145 cells to measure decatenation activity of TOPO IIα. As seen in Fig. 5c, VPA treated cells decatenated kinetoplast DNA more efficiently than untreated control cells. This suggests that although TOPO IIα is downregulated at the transcript level upon HDAC inhibition, there exists a post translational regulation whereby the protein is stabilized upon HDAC inhibition.

### Recruitment of key HR DNA repair proteins is affected upon HDAC inhibition leading to a decrease in HR DNA repair

Spatial and temporal recruitment of mediator and DNA repair proteins to the irradiation induced foci is necessary for efficient DNA repair to occur [24]. We investigated whether a decrease in DNA damage response and repair proteins upon HDAC inhibition also led to a decrease in recruitment of DNA repair proteins to the damaged site. VPA treated DU-145 and LNCaP cells were irradiated with 4 Gy of radiation and subsequently fixed after four hours of repair.

**Figure 6:**
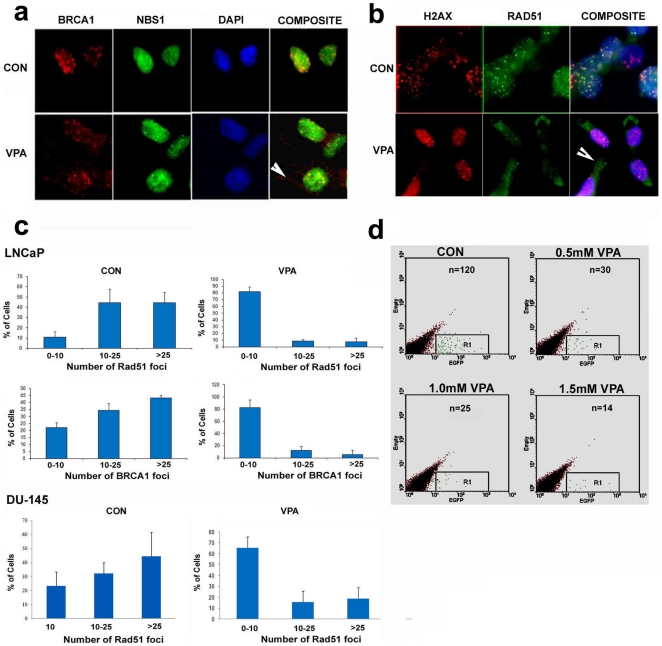
Recruitment of HR repair proteins to the damaged site and HR repair in VPA treated PCa cells. a) Immunofluorescence analysis of DU-145 cells treated with 1.5 mM VPA for 48 h and irradiated with 4Gy of radiation probed for BRCA1 (red) and NBS1 (green). Nuclei were counterstained with DAPI. Cytoplasmic BRCA1 (arrow) seen in VPA treated cells suggest impaired recruitment of BRCA1 in VPA treated cells. b) Immunofluorescence analysis of LNCaP cells treated with 1.5 mM VPA for 48 h and irradiated with 4Gy of radiation probed for H2AX Ser^139^ (red) and RAD51 (green). Nuclei were counterstained with DAPI. Cells having >25 H2AX Ser^139^foci were analyzed. Cytoplasmic RAD51 (arrow) seen in VPA treated cells suggest impaired recruitment of BRCA1 in VPA treated cells. c) Quantification of the number of BRCA1 and RAD51 foci colocalizing with H2AX Ser^139^ foci in DU-145 and LNCaP cells after treatment with 1.5 mM VPA and irradiation with 4Gy of radiation. A total of 100 cells having >25 H2AX Ser^139^foci were counted. Error bars indicate standard deviation from mean. d) FACS analysis depicting a HR repair assay using a plasmid reporter construct in LNCaP cells after treatment with varying concentration of VPA.

Immunofluorescence was carried out for BRCA1 and RAD51 along with phosphorylated H2AX proteins. There was a marked reduction in the staining for BRCA1 and RAD51 foci upon VPA treatment; control cells on the other hand showed discrete BRCA1 and RAD51 foci that colocalized with phosphorylated H2AX (Fig. 6a and b). The staining and localization of NBS1, however, remained unchanged after VPA treatment (Fig. 6a). We quantified the number of BRCA1 and RAD51 foci by counting them in cells having more than twenty H2AX foci. As shown in figure 6c, we found that there was a significant reduction in the number of repair foci for both of the repair proteins upon VPA treatment. Both LNCaP and DU-145 cells showed punctate cytoplasmic staining for both RAD51 and BRCA1. These results indicate that besides downregulation, there is an impaired recruitment of the repair proteins to the damaged site upon HDAC inhibition.

Whether this leads to a decrease in HR repair was the next question we investigated. For this, we employed a plasmid based approach to score for HR efficiency in LNCaP cells. We generated an EGFP recombination reporter construct by cloning a promoterless EGFP upstream of the pEGFPN1 vector. A Bcl I site was engineered in this gene to induce DSBs. The EGFP gene ahead of the CMV promoter was mutated, with the consequence that the functionality of EGFP would only be restored when there is efficient HR repair. As shown in the figure 6d, there was a significant reduction in the number of HR proficient LNCaP cells upon VPA treatment. These results clearly indicate an involvement of the HR pathway in PCa cells upon HDAC inhibition.

### E2F1 is involved in downregulation of key repair proteins upon HDAC inhibition

**Figure 7:**
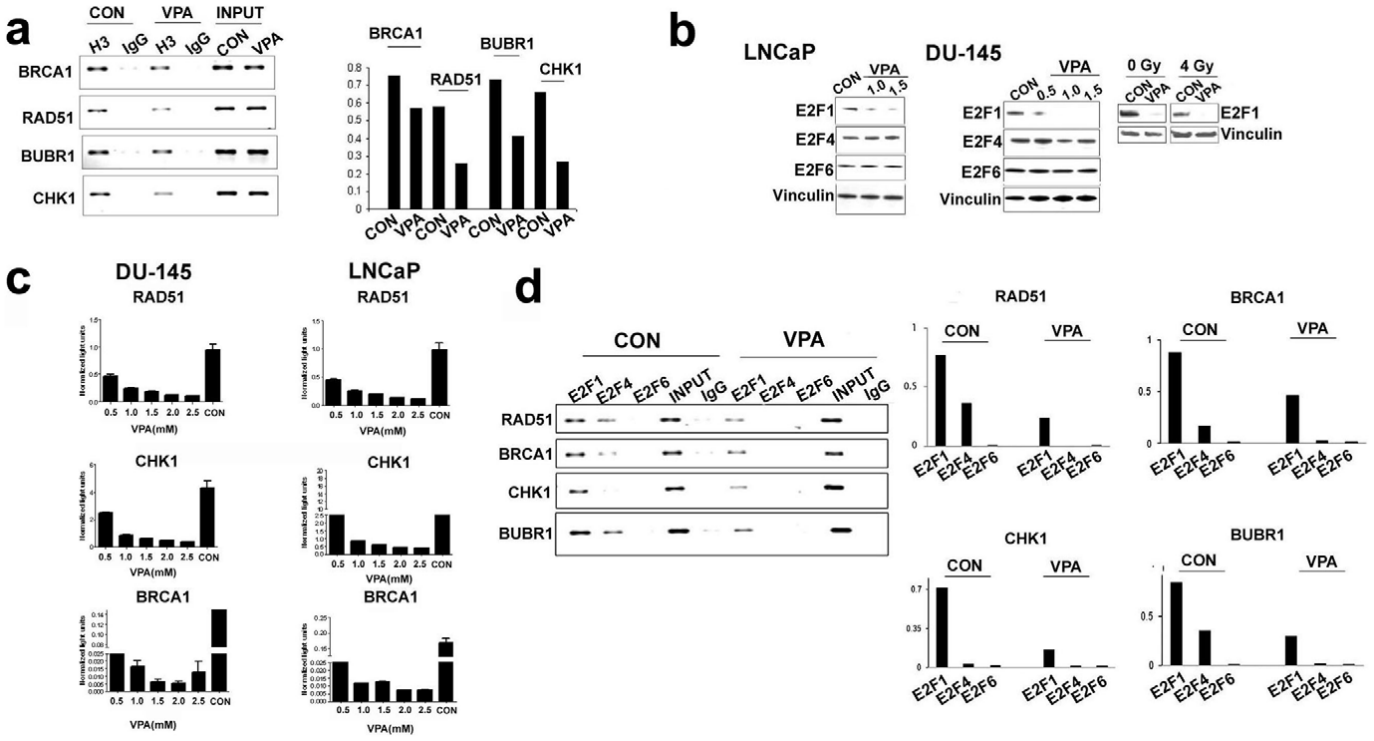
Downregulation of E2F1 mediates downregulation of DNA damage and response genes. a) ChIP analysis of VPA (1.5 mM for 48 h) treated DU-145 cells for acetylated histone H3 status in the promoters of repair genes. The bar diagram is a densitometry reading of the agarose gel shown normalized to inputs. b) Activator and repressor E2F protein levels in VPA (1.5 mM for 48 h) treated cells LNCaP and DU-145 cells.E2F levels remained downregulated upon VPA treatment even after irradiation with 4Gy radiation as shown in DU-145 cells, unirradiated (0Gy) cells served as a radiation control. c) Luciferase reporter assays in DU-145 and LNCaP cells, treated with varying concentrations of VPA, using proximal promoter regions, encompassing E2F binding regions of downregulated repair genes. d) ChIP analysis for E2F occupancy in the promoter regions of downregulated genes. ChIP was performed using antibodies against E2Fs (1, 4, and 6) in VPA (1.5 mM for 48 h) treated and control DU-145 cells. The bar diagram represents densitometric readings normalized to respective inputs.

Since our data demonstrated transcriptional downregulation of repair genes upon HDAC inhibition, we investigated the histone H3 acetylation status of promoters of a subset of downregulated genes using ChIP assays. Intriguingly, our results revealed a decrease in H3 acetylation status of the proximal promoter regions of all the genes investigated (Fig. 7a). A decrease in acetylation of promoter regions is also accompanied by an increase in binding of transcriptional repressor proteins to the promoters [7]. To investigate transcription factors that can bring about transcription repression of DNA repair genes upon HDAC inhibition, we focused our attention on the E2F transcription factors. Brca1 and Rad51 have two E2F binding site in the proximal promoter region [31]. Both Brca1 and Rad51 are repressed under hypoxic conditions by recruitment of E2F4/p130 transcription repressors. Under hypoxic conditions, E2F1 and E2F4 simultaneously bind the Brca1 promoter at two adjacent E2F sites to bring about transcriptional repression of BRCA1 gene expression [30], [31]. Similarly, Chk1 and BubR1 have transcription binding sites for E2F transcription factors and are induced by E2F1[32], [38].

Since E2F1 transcript was downregulated by HDACis, we hypothesized that HDAC inhibition may increase the binding of repressive E2Fs to downregulated repair gene promoters, and thereby result in active repression of DNA repair genes. We first investigated protein levels of both the activator and repressive E2Fs after treatment with VPA in both LNCaP and DU-145 cells. In accordance with the transcript levels, the E2F1 protein level was downregulated upon VPA treatment, while the repressive E2Fs (E2F4 and 6) did not change. E2F1 remained downregulated even after induction of DNA damage by irradiation (Fig. 7b). Thus there is a differential response of the activator and repressive E2Fs to HDAC inhibition. There are reports that suggest an increase in E2F1 transcriptional activity during neuronal apoptosis upon HDAC inhibition [39]. This could be tissue-dependent, as vorinostat mediated HDAC inhibition results in downregulation of E2F related genes in multiple myeloma [40]. Further, binding of E2F1 to the ARHI promoter was shown to be reduced by the HDAC inhibitor trichostatin A [41]. Although we found E2F1 protein levels downregulated upon HDAC inhibition, there was a possibility that the activity of the remaining pool of E2F1 was increased after HDAC inhibition. To investigate this, we cloned proximal regions of Brca1, Rad51, and Chk1, which encompasses the E2F binding sites, into the pGL3 basic luciferase reporter vector. VPA treated DU-145 and LNCaP cells were transfected with the promoter reporter constructs along with control Renilla luciferase vector. Lysates were subjected to a dual- luciferase assay. Our data revealed a significant downregulation of all the gene promoters upon VPA treatment, with the BRCA1 promoter being the most affected, as compared to control cells (Fig. 7c). Whether this involves decreased binding of E2F1 or increased binding of repressive E2F4 or E2F6 to the downregulated gene promoters was the next question we addressed. Primers for ChIP assays were designed to flank the E2F sites in proximal promoters of Brca1, Rad51, Chk1 and Bubr1 genes. Chromatin from VPA treated and untreated control cells was immunoprecipitated using antibodies against E2F1, E2F4 and E2F6. PCR amplification of precipitated chromatin DNA revealed promoter occupancy of these transcription factors. Corroborating a previous report, we did find simultaneous occupancy of E2F1 and E2F4 to Brca1 and Rad51 gene promoters and found a similar pattern for the Bubr1 promoter. Under our experimental conditions, we did not find any E2F6 binding to any of the promoters investigated. While E2F1 bound strongly to promoter regions in untreated controls, there was a significant reduction of E2F1 recruitment upon HDAC inhibition (Fig. 7d). Contrary to our hypothesis, we found that the downregulation of the repair genes is not as a result of active repression by repressive E2Fs, but an overall decrease in the recruitment of activator E2F1 to the promoters.

## Discussion

During the evolution of PCa, certain DNA repair pathways are inactivated, as a result of which, PCa acquires genomic instability. This accounts for a greater level of endogenous DNA damage in PCa cells than normal cells [42], [43]. For continued cell survival, other DNA damage response and repair pathways are induced and maintained. Therapeutics aimed at disabling remaining DNA repair pathways in PCa together with appropriate DNA damage inducers, may provide an effective therapy. Experimental evidence suggests HDACi can act as radiosensitizers for a wide variety of cancers including PCa [44]. Downregulation of repair genes has been implicated in this process. Specifically, Ku70, Ku86, and the DNAPK catalytic subunit were shown to be downregulated in melanoma cells, while Rad51 and DNAPK were downregulated in PCa cells upon HDAC inhibition [20], [45]. However, functional evaluation of specific DNA repair pathways upon HDAC inhibition has not been demonstrated. Moreover, the mechanism behind downregulation of these repair genes has not been elucidated.

We applied AFA on a microarray data set from PCa cells treated with HDACis. Our protein-protein interaction analysis revealed downregulation of several DNA repair genes that were related to BRCA1. Many of these genes are involved in the HR DNA repair pathway. The HR pathway is mediated by BRCA1 and executed by the RecA homolog Rad51 DNA recombinase, a key protein in the HR pathway [23]. Both these proteins were downregulated on HDAC inhibitor treatment. Genes of the NHEJ DNA repair pathway remained unaffected. We quantified the expression of a subset of downregulated genes both at the transcript and protein level. Downregulation after HDAC inhibitor treatment was also noted at both levels, even in the presence of radiation as a DNA damaging agent. Intriguingly, TOPO IIα, which was downregulated at the transcript level, was stabilized at the protein level. An increase in the protein level correlated with an increase in decatenation acitivity. While TOPO IIα is essential for cell survival and viability, increased activity leads to an increase in DSBs and fragmentation of DNA. This, coupled with a decrease in repair proteins upon HDAC inhibition, can potentially lead to an increase in unrepaired DNA breaks leading to a decrease in cell survival.

As predicted, treatment of PCa cells with a combination of HDAC inhibitor and DNA damaging agents like radiation, cisplatin and hydroxyurea lead to decreased survival. We attempted to understand the mechanism behind this increased sensitivity to DNA damaging agents upon HDAC inhibition. We found that besides downregulation there is an impaired recruitment of key HR repair proteins (BRCA1 and RAD51) to the DNA DSBs. Punctuate cytoplasmic staining was observed for these repair proteins upon VPA treatment. Cytoplasmic retention of both BRCA1 and RAD51 has also been reported in fibroblasts upon FGF stimulated induction of AKT1, which results in a decrease in HR DNA repair. We do not know whether cytoplasmic BRCA1 and RAD51 observed upon VPA treatments is as a result of active nuclear export of BRCA1 and RAD51 upon HDAC inhibition, or as a result of cytoplasmic retention of these proteins en-route to the nucleus. We assessed the functional consequence of downregulation and impaired recruitment of these HR repair proteins by a HR repair assay. We found a marked reduction in HR DNA repair capacity of PCa cells upon HDAC inhibition.

Recent studies have highlighted the importance of post translational histone modifications in transcriptional regulation of genes. Our study indicated a decrease in activator H3 acetylation marks in the promoter regions of the downregulated genes. How certain gene promoters maintain low histone acetylation status in the presence of HDACis is intriguing. One simple explanation could be that certain HDACs that are present in corepressors complexes are less susceptible to inhibition by HDACis, and are actively involved in repression of certain gene promoters. This has been noted in downregulation of DNMT1 by the HDAC inhibitor apicidin [17]. Recent studies indicate that the repressive E2F transcription factors can mediate or maintain the repressive histone modification, while activation histone marks are important in recruitment of activator E2Fs (E2F1-3) [46]. In order to understand the mechanism behind downregulation, we focused our attention on the E2F transcription factors. AFA indicated that E2F1 regulated genes were affected upon HDAC inhibition. Several DNA repair and response genes that were downregulated in our data set, such as Brca1, Rad51, Chk1, and Bubr1, are E2F targets. We observed that downregulation of DNA repair genes was not as a result of active repression by recruitment of repressive E2Fs to downregulated gene promoters, but was as a result of decreased recruitment of activator E2F1. Currently we are investigating whether this downregulation is through the active recruitment of HDACs or a decreased recruitment of HATs to the downregulated promoters.

In summary, our study indicates that several genes in the DNA repair pathway are affected upon HDAC inhibition. HR DNA repair is compromised upon HDAC inhibition, owing to both downregulation of HR repair proteins, and impaired recruitment of these proteins to the damaged site. Downregulation of the repair genes is on account of a decrease in amount and promoter recruitment of the E2F1 transcription factor. Since HDAC inhibition affects several pathways that could potentially have an impact on DNA repair, compromised DNA repair upon HDAC inhibition could also be attributed to several other pathways besides the ones investigated in this study. However, our study does provide insights into the mechanism that governs downregulation of HR DNA repair genes upon HDAC inhibition which can lead to rationale usage of HDACis in the clinics.

## Materials and Methods

### Cell culture and treatment

DU-145 and LNCAP PCA cell lines were obtained from ATCC and maintained in RPMI-1640 (Invitrogen) media supplemented with 10% fetal bovine serum. The cells were grown in a humidified incubator at 37°C in a 5% CO_2_ atmosphere. A stock solution of 1 M of sodium salt of VPA (Sigma, St. Louis,MO) dissolved in complete RPMI media was used for all the experiments. Cisplatin (Sigma, St. Louis,MO) and hydroxyurea (Sigma, St. Louis,MO) were dissolved in water.

### AFA Analysis

Microarray design and experimentation analysis may be obtained from our recently published manuscript (Kortenhorst et al., 2008). Functional Gene Sets were obtained from a number of different genomic databases, encompassing distinct functional themes, including Gene Ontology (GO) [47], the Kyoto Encyclopedia of Genes and Genomes (KEGG) [48], the Molecular Signatures Database [49], the UCSC Genome Browser database [50], the Stanford Microarray Database (SMD) [51], and the NCBI Entrez Gene database. Enrichment analysis was performed using a one-sided Wilcoxon rank-sum test, separately for up- and down-regulation, after ranking the genes by their signed or absolute moderated t-statistics as previously described [52]. Multiple testing correction was performed to adjust the p-values using the Benjamini and Hochberg method [53]. All such computation analyses were performed using R/Bioconductor functions and packages [54].

### Real-time quantitative PCR (Q-PCR) analysis

Cells were lysed with Trizol (Invitrogen) and total RNA was extracted. One microgram of total RNA was reverse transcribed using Superscript reverse transcriptase (Invitrogen). One tenth of the first strand cDNA reaction was used for Q-PCR amplification. Q-PCR was performed in an iCYCLER real-time PCR machine (Biorad) using SYBR-Green chemistry (BioRad). Test gene Ct values were normalized to Ct values of the house keeper gene HPRT and fold differences, as compared to untreated controls, were calculated.

### DNA Damaging Agents and Clonogenic Survival Assay

DU-145 cells were treated with varying concentrations of VPA for 48 h; untreated cells were used as controls. After treatment, cells were trypsinized and diluted to the appropriate cell density in 100-mm culture dishes to yield at least 50 colonies per dish following irradiation. Dishes were then irradiated at 0.78 Gy/min to the desired dose using a Gammacell 40 137Cs irradiator. Ten to 14 days after irradiation, colonies comprising at least 50 cells were counted after staining with crystal violet. Cell survival was plotted as a function of dose. Clonogenic assays for cisplatin and hydroxyurea was performed by treating DU-145 cells with VPA in combination with the above agents; untreated cells served as controls. Forty eight hours after treatment, cells were trypsinized and plated in 100-mm culture dishes to yield at least 50 colonies per dish. Colonies were stained and visualized as above.

### Comet Assay

Prostate cancer cells were treated with 1.5 mM VPA for 48 h before being irradiated with 6 Gy of radiation to induce double strand breaks. At different intervals of repair cells were trypsinized and subjected to an neutral comet assay using the Trevigen Comet assay kit following the manufacturer's protocol. Cells were imaged using the Zeiss Axio Scope fluorescent microscope. Comet tails were measured using the CometScore software and quantified. At least a total of fifty cells were measured per timepoint.

### Immunofluorescence and Repair Foci Recruitment Studies

Cover slip cultures of DU145 and LNCaP cells were treated with 1.5 mM VPA for 48 h; untreated cells served as controls. After 48 h cells were irradiated at 0.78 Gy/min to the desired dose using a Gammacell 40 137Cs irradiator. The cells were allowed to repair for 4 h and then fixed and permeabilized with 3.7% paraformaldehyde in PBS containing 0.125% Triton X-100 for 30 min. Fixed cells were washed and blocked for 1 h in blocking buffer (1% BSA in PBS) and probed with primary antibodies against phosphorylated H2AX Ser^139^ (mouse monoclonal, Upstate Biotechnology), NBS1 (rabbit polyclonal, Novus), Rad51 (mouse monoclonal, Abcam) and BRCA1 (mouse monoclonal, Ab-1, Calbiochem) at their respective dilutions made in blocking buffer. The cells were washed with PBS and probed with Alexa Fluor conjugated secondary antibodies (Molecular Probes). The nuclei were counterstained with DAPI. H2AX foci suggestive of DSBs was visualized under a fluorescent microscope and one hundred cells were counted and graded depending on the number of foci per cell. To investigate recruitment of repair proteins, cells with >25 H2AX foci were included. BRCA1 and RAD51 foci that colocalized with H2AX foci, suggestive of efficient recruitment, were counted and grouped.

### Immunoblotting

Cells were washed using 1X PBS and lysed on ice using RIPA buffer containing 1X protease inhibitors (Roche) and 1X PhosphoStop (Roche). 10–20 µg of protein was separated on a 4–15% SDS-PAGE gradient gel (Biorad). The separated proteins were transferred onto a PVDF (Biorad) membrane. The membrane was blocked in blocking buffer [100 mMTris-HCl pH 7.4, 0.1% Tween20, 150 mM NaCl and 5%(w/v) nonfat milk] for 1 h and incubated with primary antibodies in blocking buffer at 1∶4000 dilution, except for BRCA1 and Rad51 antibodies which were used at 1∶1000 dilution. The membrane was developed with ECL chemiluminescence (Amersham Pharmacia) after incubation with appropriate secondary antibody diluted to 1∶5000 in the blocking buffer. Band intensity was normalized with actin or vinculin as a loading control and quantified using VersaDoc gel documenting system (Biorad).

### TOPO IIα Activity Assay

Nuclear extract from VPA treated and control cells were prepared for assaying TOPO IIα activity. Five microgram of nuclear extract was used to assay TOPO IIα activity by the Eukaryotic Topoisomerase IIα Assay Kit (Topogen).

### HR Repair Assay

A plasmid based reporter assay was designed to score for HR DNA repair efficiency after VPA treatment. The EGFP gene in the pCMVEGFPN1 (Clontech) vector was mutated by a single base pair insertion that changed the codon from GGA to TGA. A promoter-less wild-type EGFP was PCR amplified using primers that had a PciI site engineered in both the forward and reverse primers, a BclI site was engineered ahead of the PciI site in the reverse primer. The PCR fragment was purified and cloned in the BspLU11I site of the pCMVEGFPN1mutant vector. A DBS was introduced by digestion with BclI and the digested vector was purified and transfected in control and VPA treated LNCaP cells, 24 h post-treatment. Twenty-four hour post-transfection green cells that had efficient HR repair were scored by flow cytometry. A control pCMVEGFPN1 (Clontech) vector, to monitor transfection efficiency, was also included.

### Promoter Reporter Assay

Proximal promoter regions of Rad51, Brca1, and Chk1 that harbor E2F sites were amplified by PCR and cloned into the pGL3 Basic luciferase reporter vector (Promega). DU-145 and LNCaP cells were treated with varying concentrations of VPA for 24 h and the above reporter constructs were transfected along with a renilla luciferase control vector in a 9∶1 ratio respectively. Twenty-four hours post transfection, cells were lysed and twenty microliters were used to perform a luciferase assay using a dual luciferase reporter assay kit (Promega). Light units were counted using a luminometer and normalized to renilla luciferase activity.

### Chromatin Immunoprecipitation (ChIP) Analysis

Forty-eight hours post-treatment with VPA, cells were treated with formaldehyde (1% final concentration) and chromatin was crosslinked for 15 min at 37°C. Crosslinking reactions were terminated by the addition of 125 mM glycine for 5 min at room temperature. Cells were washed with PBS, lysed, and chromatin was solubilized to desired length by sonication. Immunoprecipitation of crosslinked proteins was performed using acetylated H3 antibody (Upstate Biotech), E2F1 (Millipore), E2F4 (Millipore), and E2F6 (Santacruz). Isotype specific antibody processed soluble chromatin was maintained as a ChIP assay control. Immunoprecipitated DNA was analyzed by PCR using Brca1, Rad51, Bubr1 and Chk1 promoter specific primers flanking the E2F binding sites.

## References

[1] Rando OJ (2007). Global patterns of histone modifications.. Curr Opin Genet Dev.

[2] Jones PA (2002). DNA methylation and cancer.. Oncogene.

[3] Fazzari MJ, Greally JM (2004). Epigenomics: beyond CpG islands.. Nat Rev Genet.

[4] Marks P, Rifkind RA, Richon VM, Breslow R, Miller T (2001). Histone deacetylases and cancer: causes and therapies.. Nat Rev Cancer.

[5] Sparmann A, van Lohuizen M (2006). Polycomb silencers control cell fate, development and cancer.. Nat Rev Cancer.

[6] Esteller M (2007). Cancer epigenomics: DNA methylomes and histone-modification maps.. Nat Rev Genet.

[7] Denslow SA, Wade PA (2007). The human Mi-2/NuRD complex and gene regulation.. Oncogene.

[8] Shabbeer S, Carducci MA (2005). Focus on deacetylation for therapeutic benefit.. IDrugs.

[9] Weichert W, Roske A, Gekeler V, Beckers T, Stephan C (2008). Histone deacetylases 1, 2 and 3 are highly expressed in prostate cancer and HDAC2 expression is associated with shorter PSA relapse time after radical prostatectomy.. Br J Cancer.

[10] Milne JC, Denu JM (2008). The Sirtuin family: therapeutic targets to treat diseases of aging.. Curr Opin Chem Biol.

[11] Shabbeer S, Kortenhorst MS, Kachhap S, Galloway N, Rodriguez R (2007). Multiple Molecular pathways explain the anti-proliferative effect of valproic acid on prostate cancer cells in vitro and in vivo.. Prostate.

[12] Xia Q, Sung J, Chowdhury W, Chen CL, Hoti N (2006). Chronic administration of valproic acid inhibits prostate cancer cell growth in vitro and in vivo.. Cancer Res.

[13] Walton TJ, Li G, Seth R, McArdle SE, Bishop MC (2008). DNA demethylation and histone deacetylation inhibition co-operate to re-express estrogen receptor beta and induce apoptosis in prostate cancer cell-lines.. Prostate.

[14] Valentini A, Biancolella M, Amati F, Gravina P, Miano R (2007). Valproic acid induces neuroendocrine differentiation and UGT2B7 up-regulation in human prostate carcinoma cell line.. Drug Metab Dispos.

[15] Inoue K, Kobayashi M, Yano K, Miura M, Izumi A (2006). Histone deacetylase inhibitor reduces monocyte adhesion to endothelium through the suppression of vascular cell adhesion molecule-1 expression.. Arterioscler Thromb Vasc Biol.

[16] Kortenhorst MS, Zahurak M, Shabbeer S, Kachhap S, Galloway N (2008). A multiple-loop, double-cube microarray design applied to prostate cancer cell lines with variable sensitivity to histone deacetylase inhibitors.. Clin Cancer Res.

[17] You JS, Kang JK, Lee EK, Lee JC, Lee SH (2008). Histone deacetylase inhibitor apicidin downregulates DNA methyltransferase 1 expression and induces repressive histone modifications via recruitment of corepressor complex to promoter region in human cervix cancer cells.. Oncogene.

[18] Camphausen K, Scott T, Sproull M, Tofilon PJ (2004). Enhancement of xenograft tumor radiosensitivity by the histone deacetylase inhibitor MS-275 and correlation with histone hyperacetylation.. Clin Cancer Res.

[19] Camphausen K, Tofilon PJ (2007). Inhibition of histone deacetylation: a strategy for tumor radiosensitization.. J Clin Oncol.

[20] Chinnaiyan P, Vallabhaneni G, Armstrong E, Huang SM, Harari PM (2005). Modulation of radiation response by histone deacetylase inhibition.. Int J Radiat Oncol Biol Phys.

[21] Jung M, Velena A, Chen B, Petukhov PA, Kozikowski AP (2005). Novel HDAC inhibitors with radiosensitizing properties.. Radiat Res.

[22] Adimoolam S, Sirisawad M, Chen J, Thiemann P, Ford JM (2007). HDAC inhibitor PCI-24781 decreases RAD51 expression and inhibits homologous recombination.. Proc Natl Acad Sci U S A.

[23] Jeggo PA (1998). DNA breakage and repair.. Adv Genet.

[24] Harper JW, Elledge SJ (2007). The DNA damage response: ten years after.. Mol Cell.

[25] Lee JH, Paull TT (2005). ATM activation by DNA double-strand breaks through the Mre11-Rad50-Nbs1 complex.. Science.

[26] Bakkenist CJ, Kastan MB (2003). DNA damage activates ATM through intermolecular autophosphorylation and dimer dissociation.. Nature.

[27] Rogakou EP, Pilch DR, Orr AH, Ivanova VS, Bonner WM (1998). DNA double-stranded breaks induce histone H2AX phosphorylation on serine 139.. J Biol Chem.

[28] Petrini JH (2007). Cell signaling. A touching response to damage.. Science.

[29] Iaquinta PJ, Lees JA (2007). Life and death decisions by the E2F transcription factors.. Curr Opin Cell Biol.

[30] Bindra RS, Glazer PM (2007). Repression of RAD51 gene expression by E2F4/p130 complexes in hypoxia.. Oncogene.

[31] Bindra RS, Gibson SL, Meng A, Westermark U, Jasin M (2005). Hypoxia-induced down-regulation of BRCA1 expression by E2Fs.. Cancer Res.

[32] Carrassa L, Broggini M, Vikhanskaya F, Damia G (2003). Characterization of the 5'flanking region of the human Chk1 gene: identification of E2F1 functional sites.. Cell Cycle.

[33] Treszezamsky AD, Kachnic LA, Feng Z, Zhang J, Tokadjian C (2007). BRCA1- and BRCA2-deficient cells are sensitive to etoposide-induced DNA double-strand breaks via topoisomerase II.. Cancer Res.

[34] Lee E, Oh E, Lee J, Sul D, Lee J (2004). Use of the tail moment of the lymphocytes to evaluate DNA damage in human biomonitoring studies.. Toxicol Sci.

[35] Singh NP, McCoy MT, Tice RR, Schneider EL (1988). A simple technique for quantitation of low levels of DNA damage in individual cells.. Exp Cell Res.

[36] Drake FH, Hofmann GA, Bartus HF, Mattern MR, Crooke ST (1989). Biochemical and pharmacological properties of p170 and p180 forms of topoisomerase II.. Biochemistry.

[37] Borowiec JA (2004). The toposome: a new twist on topoisomerase IIalpha.. Cell Cycle.

[38] Polager S, Kalma Y, Berkovich E, Ginsberg D (2002). E2Fs up-regulate expression of genes involved in DNA replication, DNA repair and mitosis.. Oncogene.

[39] Boutillier AL, Trinh E, Loeffler JP (2003). Selective E2F-dependent gene transcription is controlled by histone deacetylase activity during neuronal apoptosis.. J Neurochem.

[40] Mitsiades CS, Mitsiades NS, McMullan CJ, Poulaki V, Shringarpure R (2004). Transcriptional signature of histone deacetylase inhibition in multiple myeloma: biological and clinical implications.. Proc Natl Acad Sci U S A.

[41] Lu Z, Luo RZ, Peng H, Huang M, Nishmoto A (2006). E2F-HDAC complexes negatively regulate the tumor suppressor gene ARHI in breast cancer.. Oncogene.

[42] DeMarzo AM, Nelson WG, Isaacs WB, Epstein JI (2003). Pathological and molecular aspects of prostate cancer.. Lancet.

[43] Chan N, Milosevic M, Bristow RG (2007). Tumor hypoxia, DNA repair and prostate cancer progression: new targets and new therapies.. Future Oncol.

[44] Paoluzzi L, Figg WD (2004). Histone deacetylase inhibitors are potent radiation protectants.. Cancer Biol Ther.

[45] Munshi A, Kurland JF, Nishikawa T, Tanaka T, Hobbs ML (2005). Histone deacetylase inhibitors radiosensitize human melanoma cells by suppressing DNA repair activity.. Clin Cancer Res.

[46] Bracken AP, Ciro M, Cocito A, Helin K (2004). E2F target genes: unraveling the biology.. Trends Biochem Sci.

[47] Ashburner M, Ball CA, Blake JA, Botstein D, Butler H (2000). Gene ontology: tool for the unification of biology. The Gene Ontology Consortium.. Nat Genet.

[48] Kanehisa M, Goto S, Kawashima S, Okuno Y, Hattori M (2004). The KEGG resource for deciphering the genome.. Nucleic Acids Res.

[49] Subramanian A, Tamayo P, Mootha VK, Mukherjee S, Ebert BL (2005). Gene set enrichment analysis: a knowledge-based approach for interpreting genome-wide expression profiles.. Proc Natl Acad Sci U S A.

[50] Karolchik D, Baertsch R, Diekhans M, Furey TS, Hinrichs A (2003). The UCSC Genome Browser Database.. Nucleic Acids Res.

[51] Demeter J, Beauheim C, Gollub J, Hernandez-Boussard T, Jin H (2007). The Stanford Microarray Database: implementation of new analysis tools and open source release of software.. Nucleic Acids Res.

[52] Daniel VC, Marchionni L, Hierman JS, Rhodes JT, Devereux WL (2009). A primary xenograft model of small-cell lung cancer reveals irreversible changes in gene expression imposed by culture in vitro.. Cancer Res.

[53] Benjamini Y, Drai D, Elmer G, Kafkafi N, Golani I (2001). Controlling the false discovery rate in behavior genetics research.. Behav Brain Res.

[54] Gentleman RC, Carey VJ, Bates DM, Bolstad B, Dettling M (2004). Bioconductor: open software development for computational biology and bioinformatics.. Genome Biol.

